# A Novel Ergonomic Curette Design Reduces Dental Prophylaxis-Induced Muscle Work and Fatigue

**DOI:** 10.3390/dj11120272

**Published:** 2023-11-28

**Authors:** Kairong Lin, Cherie Wink, Ben Dolan, Kathryn Osann, Ali A. Habib, Jill Gehrig, Petra Wilder-Smith

**Affiliations:** 1 Beckman Laser Institute, University of California Irvine, Irvine, CA 92612, USA; kaironl@uci.edu (K.L.); cwink@hs.uci.edu (C.W.); 2 Henry Samueli School of Engineering, University of California Irvine, Irvine, CA 92617, USA; dolanb@uci.edu; 3 School of Medicine, University of California Irvine, Irvine, CA 92612, USA; kosann@hs.uci.edu; 4 Department of Neurology, University of California Irvine, Orange, CA 92868, USA; aahabib@hs.uci.edu; 5 Asheville-Buncombe Technical Community College, Asheville, NC 28804, USA; jill.gehrig@gmail.com

**Keywords:** adaptive curette, dental hygienist, dental hygiene, ergonomics, electromyography, musculoskeletal disorder

## Abstract

Background: To compare fatigue, comfort, and muscle work associated with the use of two periodontal curettes during scaling: one with a novel adaptive design, the other with a conventional non-adaptive design. Methods: Twelve hygienists scaled a typodont using two Universal Barnhart 5/6 curettes: (1) a prototype featuring an adaptive silicone-covered handle (Curette A), and (2) a stainless-steel curette (Curette B). Surface Electromyography (sEMG) traced muscle work. Hand positions, fatigue, comfort, pinch, and grasp strength were recorded. Paired t-tests and a repeated measures ANOVA with covariates were tested for differences. The significance level was set at *p* < 0.05. Results: Curette A performed significantly better in all categories. Pinch and grasp strength and fatigue were significantly reduced post-instrumentation for Curette B. Curette A required significantly less (i) total muscle work and (ii) work in individual muscles. Comfort, correct grasp, and blade adaptation were significantly better using Curette A. Conclusions: A curette featuring a novel adaptive handle design demonstrated significantly improved ergonomic performance. Additional clinical studies are needed to solidify our understanding of the potential short- and long-term benefits of the novel curette handle design. Practical Implications: A novel adaptive curette handle design that enables the clinician to adapt the instrument across the index finger may reduce musculoskeletal burden and fatigue, as well as improve comfort during periodontal instrumentation.

## 1. Introduction

Dental hygiene ranks first among all occupations in the US with regard to the prevalence of carpal tunnel syndrome (CTS) [1], musculoskeletal diseases (MSDs) [2,3], and upper extremity disorders [1,4,5,6,7,8,9]. Approximately 45% of dental hygienists report symptoms of CTS, and almost 10% are medically diagnosed with this debilitating disorder, which often develops within just a few years of clinical practice in these clinicians [10]. One study found that 64–96% of hygienists experienced symptoms of MSDs within a 12-month period, adversely affecting the quality of life and career longevity of these individuals [10]. Other dental professionals are commonly affected by these disorders as well [11,12,13,14,15,16,17,18], with two-thirds of all dental clinicians reporting occupational musculoskeletal pain [2,16,17,18,19,20,21,22,23,24,25,26,27]. Many dental clinicians are compelled to work part-time due to work-related MSDs, and nearly a third of dentists are forced to retire early because of this condition [18], which also adversely affects clinical effectiveness, productivity, and work satisfaction [8,12,18]. One study estimated an annual income loss of $131 million due to MSDs in the dental profession [28].

Effective manual periodontal instrumentation requires precise positioning and control of the dental tool while executing high-force, often non-axial maneuvers [29,30]. The scaling process is repetitive, involving 1–3 mm channeling strokes applied over periods of up to an hour, with typically very few rest pauses. Pinch and grasp forces during instrumentation are sustained and considerable [31,32,33]. Often these forces are maintained during extended non-neutral wrist positions while performing isotonic or isometric muscle work, which can result in damage to the hand, wrist, arm, head, neck, torso, and back [31,32,33]. The combination of repetitive, sustained work in the small muscles of the fingers and hand, the need to apply strong pressure or pulling forces over long periods, often while seated in a non-ergonomic position, and the need to perform this work without adequate breaks in order to maintain a viable business model all contribute to the health risks of manual periodontal instrumentation.

Previous studies have established that individual design features of dental instruments, including handle diameter, hardness, texture, weight, and thermal conductivity, are all important determinants of their ergonomic performance [29,34,35,36,37,38,39]. The short- and long-term musculoskeletal health of dental clinicians, as well as the clinical effectiveness of manual periodontal instrumentation by these individuals, are all affected by such design parameters. The impact of these design features has been investigated using many neurophysiological techniques. These include Electromyography (EMG) and surface Electromyography (sEMG) to evaluate muscle work at specific sites during instrumentation [29,32,36,40]. Furthermore, goniometry, inclinometry, kinematics, and accelerometry techniques have all been utilized to evaluate overall and location-specific posture as well as the movements of individual parts of the body during instrumentation [29,41,42]. Force sensors, or dynamometers, are typically used to measure pinch and grasp strength before, during, and after instrumentation. Many instrumentation processes result in a loss of muscle strength and reduced instrumentation effectiveness over time [31,35,43]. Pressure sensors mounted on periodontal instruments are able to monitor the magnitude of the grip and grasp force applied during instrumentation. These forces exert a direct influence on operator comfort, efficacy, and fatigue [31,35,43]. Moreover, there exists a considerable body of work outside the field of dentistry that addresses the role of additional variables in improving the ergonomic properties of hand tools, especially those undergoing repetitive and prolonged use. These include sewing, gardening, and workmen’s tools. Ergonomic measures include design features to spread instrumentation weight and forces over larger surface areas and over anatomical structures beyond the fingers and thumb. In this way, a lower pressure per unit area and a more favorable pressure distribution can be achieved [44,45,46,47,48,49,50]. Moreover, the area of contact between the handle and the hand and fingers can, for example, be maximized to spread work-related loading over a larger surface area of the fingers, hand, or arm [2,44,46,47,48,49,50]. Other design-focused modifications have been implemented to improve instrument access to all areas of the mouth, including poorly accessible sites such as posterior regions, while maintaining healthy hand and wrist position as well as full-body posture [2,32,51,52,53]. 

The goal of this study was to compare the fatigue, comfort, and muscle work associated with periodontal instrumentation to remove calculus in a typodont model using a novel adaptive curette design vs. a conventional non-adaptive stainless-steel curette configuration. 

## 2. Materials and Methods

This protocol was reviewed by the University of California, Irvine’s IRB and granted exempt status, as only de-identified, coded data were recorded during testing in typodont models.

### 2.1. Testers

Ten right-handed and two left-handed hygienists participated in this study. All were employed 3–5 days per week in clinical practice. Study participants were recruited by phone calls, e-mails, and text messages. Individuals with any injuries to their fingers, hands, or wrists within 6 months of study begin, and those with any symptoms or diagnosis of upper-extremity MSDs were excluded from the study to exclude any cofounding data from pre-existing conditions.

### 2.2. Protocol

**Figure 1:**
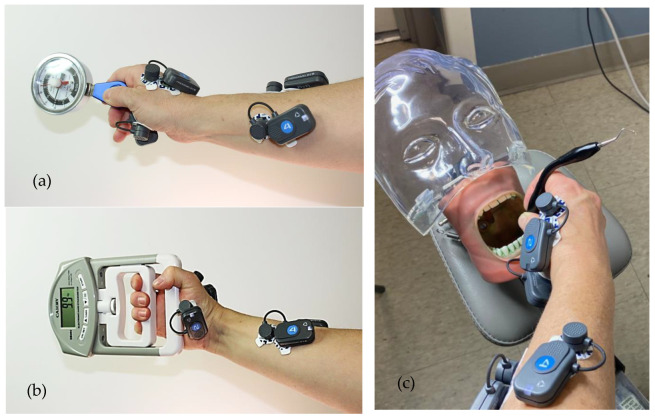
(**a**) Pinch dynamometer measurements; (**b**) Grasp dynamometer measurements; (**c**) Experimental set-up.

Artificial calculus (Dental Calculus Set, Kilgore International Inc., Coldwater, MI, USA) was applied to artificial teeth in a typodont model by the same researcher in a standardized fashion. The calculus application was timed to be completed 18 h before each study leg. Timing was important, as the artificial calculus used in this study increases in hardness over time, and equivalent hardness was necessary for each study leg and tester to ensure a comparable workload. The simulated calculus was applied supra- and sub-gingivally to 32 artificial teeth, simulating an entire dentition. The artificial teeth were subsequently mounted in standard typodont models and then attached to a manikin face (Kilgore International Inc., Coldwater, MI, USA). Finally, each manikin was attached to a clinical dental chair using a standard clamp and rod apparatus (Figure 1).

Each tester was instructed to perform scaling on the typodont teeth mounted in the mannikin as if working on a real patient, applying sufficient force to remove completely the simulated calculus deposits without damaging the surfaces of the underlying teeth. Each tester used 2 universal curettes (Barnhart 5/6), one for each study arm, with the sequence of use randomized 1:1 (https://www.randomizer.org/, accessed on 14 August 2023) and the total study duration approximating 60–90 min. One instrument was a prototype adaptive curette custom-built in our engineering and prototyping laboratory. It features a central silicon-covered handle that can be adjusted universally to any desired shape with a “bend” of up to 30° (curette A). During use, the handle remains stable and loadable in whatever shape it has been configured to, and it can be re-shaped to meet specific access or grasp parameters throughout instrumentation. The second curette used in this study was a standard rigid stainless-steel curette (curette B). Before study begin, the testers were shown a 1 min recorded instructional video on use of the adaptive curette, after which they were given 5 min to accustom themselves to the instruments. As each hygienist tested both curette A and curette B, each clinician sequentially worked on 2 typodont models, scaling each model for 8 min, with a 20 min rest period between the 2 study arms. In each study arm, 1 min was spent scaling each of 8 typodont segments, respectively, with instrumentation following the same routine for both curettes and for all testers: (1) lower anterior sextant facial surfaces; (2) lower anterior sextant lingual surfaces; (3) upper anterior sextant facial surfaces; (4) upper anterior sextant lingual surfaces; (5) lower right sextant buccal surfaces; (6) lower left sextant buccal surfaces; (7) upper right sextant buccal surfaces; and (8) upper right sextant lingual surfaces. Testers were free to adjust the typodont position and their own chair throughout the study, and they were able to work from their preferred clock positions at all times.

The duration of the rest period between the 2 study arms (using instrument A and instrument B) was elucidated in a prior pilot study to ensure that there was no carryover in fatigue from study arm 1 to study arm 2, and to ensure a return to baseline for all evaluation parameters prior to the begin of the second arm of the study. In this pilot study, sEMG and fatigue data using the same techniques as described for the definitive study were collected from 2 hygienists while they followed the study protocol using only 1 curette for 8 min. Subsequently, their sEMG and VAS values were recorded for an additional hour to identify the timepoint at which all values had returned to baseline. The 2 pilot study testers completed one such test using instrument A on each of 3 days and then again for instrument B on 3 additional days, so that any post-instrumentation effects were measured a total of 3 times for each instrument in each of the 2 testers.

Throughout instrumentation, video recordings of the testers’ hands documented instrument grasp and blade position on the tooth surface. A video camera was positioned so that the testers’ hands and fingers were in full view throughout each instrumentation episode—depending on the instrumentation location; the video camera was re-positioned as needed to ensure a full and unobstructed view of the testers’ hands. Directly at the end of each study arm, tester fatigue and comfort were recorded using conventional VAS surveys, with 0 being “no fatigue or discomfort” and 10 being “extreme fatigue or discomfort.” Pinch and grasp strength were each measured 3times before and again 3 times after each study arm using standard pinch and grip force dynamometers (Jamar, J.A. Preston Corp., Clifton, NJ, USA) (Figure 1). Finally, testers provided their spontaneous comments regarding the two curettes, and their statements were noted.

### 2.3. Instruments

**Figure 2:**
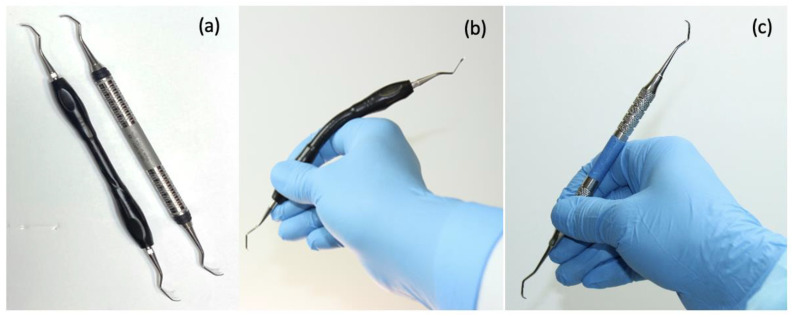
Dental curettes: (**a**) LHS: prototype adaptive curette; RHS: conventional stainless-steel curette; (**b**) prototype adaptive curette in 1 adaptive configuration; (**c**) conventional stainless-steel curette.

All instruments were configured as a universal curette (Barnhart 5/6). Two dental curettes with differing handle designs but the same working ends and similar lengths and weights were tested. (Table 1 and Figure 2). Because of the very different appearance and functionality of the two curettes tested, it was not possible to blind the testers to curette identity during instrumentation.

Curette A: a prototype curette (5/6 Barnhart) whose handle features a central flexible, universally adjustable (up to an angle of approximately 30°) core, which allows the instrument to adapt closely to the curvature of the hand and fingers during instrumentation. A silicone overlay of the handle provides a cushioned, thermally insulated grip.

Curette B: a conventional stainless-steel curette whose parameters align closely with some of the most widely used curettes (5/6 Barnhart Curette Stainless Steel, Sterling^®^, Menlo Park, Gauteng, South Africa). 

### 2.4. Surface Electromyography (sEMG) 

Using surface EMG electrodes (FREEEMG, ©BTS Engineering, Quincy, MA, USA), live continuous recordings were captured in 4 muscle locations prior to and during scaling. Muscles were selected that are specifically used for gripping and manipulating dental instruments. These muscles undergo considerable loading during dental instrumentation [1,4,29,31,33,35,36,40,42]. SEMG activity was recorded from the following muscles:

1. Abductor Pollicis Brevis (APB)
2. First Dorsal Interosseous (FDI)
3. Flexor Pollicis Longus (FPL)
4. Extensor Digitorum Communis (EDC)

Accurate electrode placement (Figure 1) was confirmed using standard muscle function tests for each site, and electrode locations were adjusted until each electrode was fully operational and optimally positioned [45]. The electric action potential signals produced during muscle action were transmitted wirelessly to a Dell laptop via a USB-port dongle that connected with proprietary software on the laptop computer (FREEEMG, ©BTS Engineering, Quincy, MA, USA). Next, testers performed maximum voluntary isometric contractions (MVC) over a period of 15 s for each muscle [53]. The resultant sEMG traces were recorded, considered 100% activity for that muscle, and hence used to normalize the subsequent sEMG data. This is a standard method that has been used during dental scaling and has been found to be reliable for use with surface electrodes [54,55,56,57]. Once baseline data collection was completed, sEMG signals from all four muscles were recorded throughout instrumentation to achieve calculus removal in the typodont models. Subsequently, raw sEMG signals were rectified and filtered using a second-order Butterworth filter with a 10 Hz high-pass cutoff frequency. Finally, total muscle activity was calculated from the integrated sEMG curve, which measures the total area under the curve (total workload) during the entire period of instrumentation for calculus removal.

### 2.5. Evaluation Criteria 

Immediately after each study arm, testers provided evaluations of the following variables:

A. Fatigue: Testers completed a standard VAS scale that ranged from 0–10, with 0 being no fatigue and 10 being maximum fatigue in the hand.
B. Comfort in wrist, fingers, and palm: Testers completed a standard VAS scale that ranged from 0–10, with 0 being complete comfort and 10 being maximum discomfort at the wrist, fingers, and palm, respectively.
C. Pinch strength was measured 3 times before and 3 times after each study arm by means of a dynamometer. The tester was blinded during measurement.
D. Grasp strength was measured 3× before and 3× after each study arm by means of a dynamometer. The tester was blinded during the measurement.
E. Muscle work during scaling: sEMG traces were analyzed using the BTS EMG analyzer^TM^ software (FREEEMG, ©BTS Engineering, Quincy, MA, USA).
F. Using video recordings of all instrumentation by each clinician, one pre-standardized evaluator with more than 20 years of experience in dental hygiene and instrumentation expertise evaluated the following variables:
  (a) Clinicians’ grasp:
    (a1) Finger positioning, including finger pad, index, and middle finger placement on the instrument: Correct/Incorrect
    (a2) Maintaining an ergonomically favorable C-shaped convex configuration of the index finger and thumb, using a “knuckles up” position to prevent joint hyperextension: Yes/No
    (a3) Fingertip blanching, indicative of an excessively tight grasp: Yes/No
  (b) Approximately 70-degree blade-to-tooth adaptation: Correct/Incorrect
G. Clinicians were asked to provide open-ended comments regarding the two handle designs. Additionally, participants were asked to indicate which instrument they preferred and the reasons for their preference.

### 2.6. Statistical Analysis 

Standard SPSS 19 statistics software (IBM^®^, Armonk, NY, USA) was used for data analysis. Paired t-tests were used to analyze the data. A repeated measures analysis of variance model with covariates (sequence and age/experience) was additionally used to test for differences after adjustment for covariates. The level of significance was set at *p* < 0.05. 

## 3. Results

Twelve hygienists, eleven female and one male, aged 24–68 (mean age 36.1 years, median age 35), were recruited as instrument testers in this study. Three of the hygienists had 2–5 years of clinical experience, three had 6–10 years of clinical experience, and six had worked for 11 or more years in clinical practice. The testing session was completed in full compliance with the protocol by all 12 testers.

Testing results were not significantly affected by testers’ duration of clinical practice for any of the evaluation criteria (*p* > 0.05). The instrumentation sequence and left vs. right-handedness also did not significantly affect any of the evaluation criteria (*p* > 0.05).

### 3.1. Comfort and Fatigue (Figure 3)

**Figure 3:**
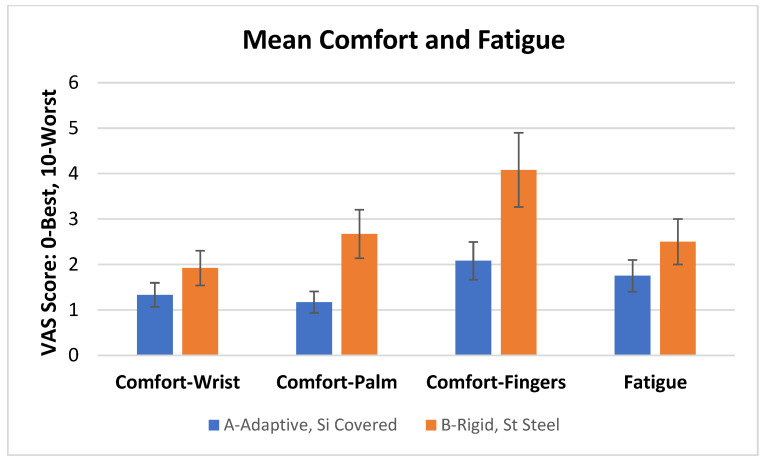
Mean comfort and fatigue scores (S.D.) for each curette. Adaptive curette A performed significantly better than curette B (*p* < 0.05) in all categories.

Adaptive curette A performed significantly better than curette B (*p* < 0.05) in all categories, with hygienists reporting better comfort in the hand, wrist, and fingers, as well as less overall fatigue when using the prototype adaptive curette (Figure 3).

### 3.2. Pinch Force and Grasp Force (Figure 4)

**Figure 4:**
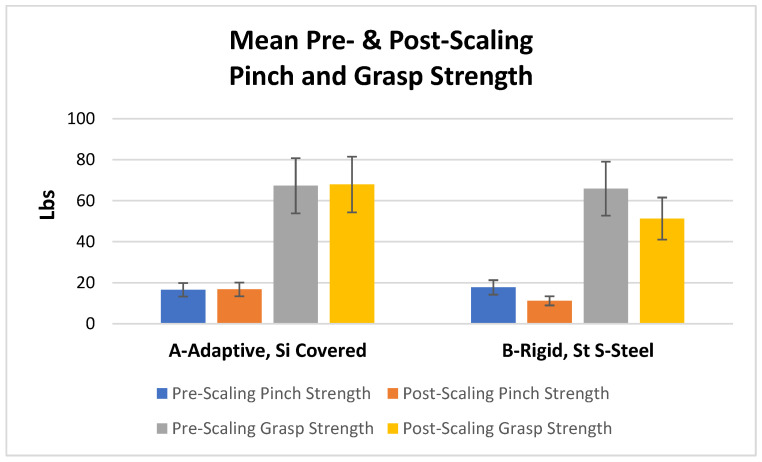
Mean pre- and post-scaling pinch and grasp strength (S.D.) for each curette. Pinch and grasp strength were significantly reduced after instrumentation with curette B but not after use of curette A (*p* < 0.05).

Pre-scaling pinch and grasp strengths did not differ significantly between the two instrument types (*p* > 0.05). Pinch and grasp strength were significantly reduced after instrumentation with curette B but not after use of curette A (*p* < 0.05) (Figure 4).

### 3.3. Surface EMG Data (Figure 5)

**Figure 5:**
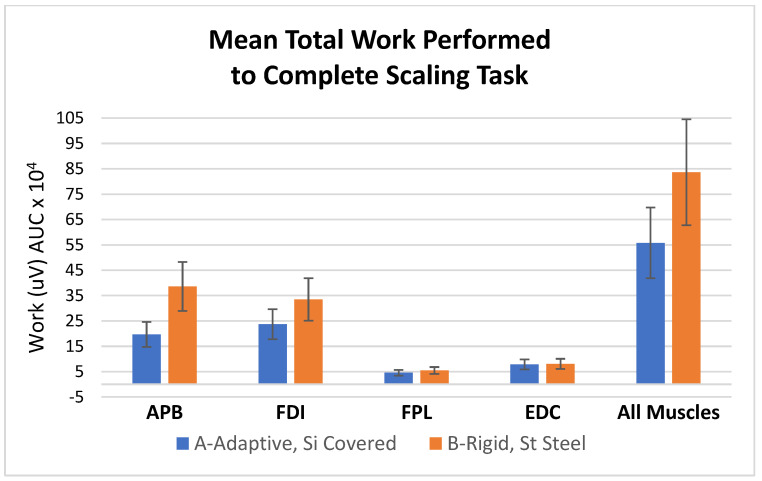
Mean total work (S.D.) performed to complete the standardized scaling task. APB-abductor pollicis brevis; FDI-first dorsal interosseous; FPL-flexor pollicis longus; EDC-extensor digitorum communis. Curette A required significantly less (i) total muscle work and (ii) work in each individual muscle for completing the set scaling task than curette B (*p* < 0.05).

Curette A required significantly less (i) total muscle work and (ii) work in each individual muscle for completing the set scaling task than curette B (*p* < 0.05) (Figure 5).

### 3.4. Video-analysis of Curette Grasp and Adaptation

During scaling, all clinicians maintained a correct modified pen grasp, with the pads of the thumb and index finger correctly opposed to each other. The hygienists’ middle finger pads remained in close proximity to the lower shank of each curette throughout this study. Hypermobility (joint hyperlaxity or hyperextension) occurred significantly more often using curette B than using curette A (*p* < 0.05) (Table 2).

Blade positioning—with the terminal shank parallel to the long axis of the tooth—was significantly better using curette A than curette B (*p* < 0.05). Blade adaptation to the tooth surface was also significantly better using curette A (*p* < 0.05), with a correct angle of 70° achieved twice as often with curette A vs. curette B (Table 3).

### 3.5. Tester Comments

One tester requested additional training in the use of curette A prior to study begin and after watching the instructional video. Another tester requested an additional 5 min at study begin to accustom herself to curette A. At the end of this study, two clinicians reported that curette A felt overall more comfortable in their hands before and during instrumentation than curette B. Two clinicians noted that curette A was more comfortable and caused less fatigue than curette B during instrumentation of the anterior sextants. These testers also felt that, using curette A, forces and pressure from instrumentation were spread across a larger area, reducing finger and thumb strain and fatigue as compared with curette B. Two testers commented on improved instrument access throughout the oral cavity using curette A vs. curette B. The same individuals also reported better blade adaptation to the lingual surfaces of the lower anterior teeth using curette A. Five testers reported a sensation of using lower pinch and grasp forces during instrumentation with curette A vs. curette B. One clinician described the silicon handle covering of curette A as too “rubbery” and would have preferred a slightly stiffer surface.

## 4. Discussion

In this study, the ergonomic performance of two curette handle designs was compared. The experimental design was based on our prior comprehensive analysis of pertinent variables in the ergonomic performance of hand instruments, especially dental ones. Because this is our first study on this topic, we were unable to test all variables at once, and we are currently conducting additional extensive studies that examine other variables and compare prototype performance with a wider range of existing designs. For this study, the prototype was compared against a stainless steel instrument with a smaller diameter than the prototype because the dimensions of the commercial instrument echo those of some of the most popular and well-established curette brands. We recognize that the difference in performance between the prototype and the commercial instrument is likely the cumulative result of many design differences beyond the adaptive component, including instrument diameter and surface covering [2,32,36,51,52,58]. Similarly, we recognize that an evaluation of the effect of the design changes on curette performance in terms of scaling duration and outcomes is important, and this variable is also included in current ongoing testing.

The potential role of the testers’ length of clinical practice was evaluated because those with longer years in clinical practice might have more sensitive musculoskeletal systems and might have an unrecognized preference for the conventional curette handle design to which they had become accustomed. However, the hygienist’s length of clinical practice did not significantly affect any of the variables that were measured. Clinician left- vs. right-handedness, which might affect clinician posture, instrument grasp, and work patterns, also did not significantly affect any of the variables measured. The sequence of instrument use was not significant either, most likely due to the implementation of a relatively long, 20 min rest period between study arms.

It has been shown that, for reliable assessment of the ergonomic performance of a hand tool, both objective and subjective measurements are needed, as a large number of very diverse variables contribute to instrument performance and user satisfaction [44,59,60]. Thus, many studies combine objective measurements such as EMG or sEMG traces with subjective evaluations of variables such as comfort and fatigue, often using VAS tools. However, the reported findings using these measures have sometimes appeared contradictory. It is our opinion that this area of research requires further investigation and the development of better standards as well as evaluation protocols and tools [2,30,32,35,36,44,45,46,47,48,49,50,52].

In this study, there was an overall agreement between the objective measurements of work and fatigue and subjective evaluations of the same. Participants reported more comfort and less fatigue using curette A vs. curette B, which aligns well with the data from sEMG as well as pinch and grasp force measurements. Clinicians provided favorable feedback with regard to prototype performance in many regards, and there were no comments from testers with regard to tactile feedback from the silicone-covered vs. metal instrument. One clinician did not like the rubbery “feel” of the silicone. Testers were unable to wear gloves during this study as they would have interfered with the sEMG electrodes, and additional studies are underway using a different study design that allows clinicians to wear gloves during instrumentation. Although the prototype featured a silicone surface with a similar Shore scale hardness to the leading commercial silicone instruments, we also plan to compare the quality of the tactile feedback of the two handle materials in future studies.

The prototype adaptive curette handle tested in this study was designed to address multiple variables that have been shown to have a positive impact on the ergonomic performance of repetitively used hand tools. A design that ensures maximum contact between the instrument handle and the hand and fingers allows for an efficient transfer of forces from the operator to the instrument and avoids ineffective work by the clinician [2,44,45]. The adaptive design of the prototype instrument achieves this goal by allowing the handle to adapt more closely than linear instruments to the shape of the fingers and hand during use. This feature likely contributed considerably to the reduced amount of work by the clinician required to complete the set scaling task using the adaptive curette. Moreover, the sEMG data evidencing the lesser amount of muscle work required to complete the set scaling task with curette A vs. curette B was confirmed by the significantly lower levels of fatigue and better comfort experienced by all testers when using curette A. We hypothesize that the prototype curette reduces loading per unit area of the fingers and hand during scaling by spreading the instrumentation weight and forces over a wider area of the index finger, thus reducing stress on musculoskeletal structures. Additional studies are under way to test this concept. Previous studies have similarly reported that distributing such forces over a larger surface area is functionally and ergonomically advantageous [44,45,61], supporting the findings in this study that scaling with the adaptive curette was associated with better comfort, less fatigue, and reduced muscle work. Multiple previous studies have reported that softer hand instruments with a larger diameter are more comfortable and less stressful on the musculoskeletal system than their narrower counterparts fashioned from metal [2,32,36,51,52,58]. Indeed, there is now a trend towards softer, thicker hand instruments and tools across the board to take advantage of this concept.

Finally, video analysis and tester comments from study participants demonstrated that the adaptive design provided the clinician with improved instrument access to all areas of the mouth while maintaining a healthy finger, hand, and wrist position. This property will contribute to musculoskeletal health and clinical efficacy by reducing the need for postural misalignments during instrumentation [2,32,51,53].

In summary, a curette featuring a novel adaptive handle design demonstrated significantly improved ergonomic performance over a curette with a conventional stainless-steel handle. This first study was performed by dental hygienists working in typodont models attached to a clinical dental chair. More extensive in vivo studies are now underway in dental patients to solidify our understanding of the ergonomic performance and clinical effectiveness of this novel adaptive handle design approach.

## 5. Practical Implications and Conclusions

A periodontal curette with a novel adaptive handle design may provide ergonomic benefits, including improved comfort, less muscle work, and reduced fatigue related to periodontal scaling. Additional studies are needed to solidify these initial findings and to investigate the potential long-term benefits of this design concept to musculoskeletal health in dental clinicians and others engaging in repetitive work with hand tools.

## Figures and Tables

**Table 1:** Overview of instruments used in this study.

	Curette A	Curette B
Instrument Type	Barnhart 5/6	Barnhart 5/6
Handle Material	Silicone-covered	Stainless-steel
Curette Configuration	Adaptive	Rigid
Handle Length	104 mm	104 mm
Instrument Length	172 mm	168 mm
Handle Diameter @ Pen Grip	12.41 mm	8.43 mm
Curette Weight	14.60 g	14.65 g
Blade Material	Stainless-steel	Stainless-steel

**Table 2:** Evaluation of curette grasp for each curette: Mean % (S.D.) of 8 scaling locations in which the correct grasp position was achieved for each curette.

Criterion	Curette A	Curette B
Index finger and thumb pads remained opposed	100 (15)	80 (12)
Correct middle finger placement	100 (18)	80 (14)
Hyperextension of the index finger or thumb avoided	80 (9)	40 (5)
Correct ring finger position	100 (10)	80 (8)

**Table 3:** Evaluation of curette blade position during instrumentation: Mean (S.D.) % of 8 locations in which the correct blade position was achieved.

Criterion	Curette A	Curette B
The terminal shank is parallel to the long axis of the tooth	100 (15)	80 (12)
Blade maintained at a 60–80-degree angle to the tooth	100 (18)	80 (14)

## Data Availability

Data are available on request due to restrictions. The data presented in this study are available on request from the corresponding author. The data are not publicly available due to privacy restrictions.
